# The Promise of Novel Biomarkers for Head and Neck Cancer from an Imaging Perspective

**DOI:** 10.3390/ijms19092511

**Published:** 2018-08-24

**Authors:** Loredana G. Marcu, Paul Reid, Eva Bezak

**Affiliations:** 1 Faculty of Science, University of Oradea, 410087 Oradea, Romania; 2 Cancer Research Institute and School of Health Sciences, University of South Australia, Adelaide, SA 5001, Australia; paul.reid@mymail.unisa.edu.au (P.R.); Eva.Bezak@unisa.edu.au (E.B.); 3 Department of Physics, University of Adelaide, Adelaide, SA 5005, Australia

**Keywords:** hypoxia, proliferation, cancer stem cells, radioresistance, PET, SPECT, MRI

## Abstract

It is an agreed fact that overall survival among head and neck cancer patients has increased over the last decade. Several factors however, are still held responsible for treatment failure requiring more in-depth evaluation. Among these, hypoxia and proliferation-specific parameters are the main culprits, along with the more recently researched cancer stem cells. This paper aims to present the latest developments in the field of biomarkers for hypoxia, stemness and tumour proliferation, from an imaging perspective that includes both Positron Emission Tomography (PET) and Single Photon Emission Computed Tomography (SPECT) as well as functional magnetic resonance imaging (MRI). Quantitative imaging of biomarkers is a prerequisite for accurate treatment response assessment, bringing us closer to the highly needed personalised therapy.

## 1. Introduction

Advances in imaging and treatment technology over the last few decades have brought an improvement in locoregional control among head and neck cancers (HNC) [1]. The 5-year survival rates however, are still poor in this patient group, mainly due to treatment resistance, recurrence and distant metastasis. While treatment management of early-stage head and neck cancer is less problematic, advanced cancers impose a much higher degree of difficulty given by various factors held responsible for treatment failure, requiring more in-depth evaluation. Hypoxia and proliferation-specific parameters are the main culprits, together with the more recently researched cancer stem cells. The synergistic effect of these parameters adds further challenge to various treatment modalities in tumour resistance [2,3]. The solution to this is most likely to consist of a more personalised treatment approach.

A few decades ago, the scientific community was focusing on developing predictive assays for oxygen status, proliferation and intrinsic radioresistance in order to design efficient treatment regimens in a personalised manner. While some assays have shown promise when tested in vitro, their success in vivo was never achieved due to several limitations [4]. Table 1 is a compilation of the three main predictive assay categories, highlighting their purpose and limitations [5]. Based on predictive assays, conclusions can be made regarding the optimal treatment for an individual. Since head and neck tumours are known to be typically hypoxic, testing oxygenation levels could dictate the need for hypoxic cell sensitisers or cytotoxins. Furthermore, altered fractionation schedules based on proliferation kinetics, have shown potential to improve locoregional-control in advanced HNC patients, while combined modality treatment or other forms of radiotherapy (brachytherapy, proton therapy) are effective in patients with radioresistant tumours. Still, a more personalised patient selection is needed in order to reduce adverse events and further improve tumour control in HNC.

These initial studies on predictive assays opened new research avenues and changed the way radiation therapy is currently planned and delivered. Most changes are due to advances in knowledge on the molecular and genetic determinants of cellular response to radiation, well described by specific biomarkers. A number of tumour-specific biomarkers are currently undergoing research to be used in functional imaging and/or targeted therapies and several promising candidates are under clinical investigation worldwide.

One of the breakthrough findings in the management of head and neck cancer was the predictive role of the human papilloma virus (HPV), as patients with oropharyngeal cancer that tested positive for HPV, had significantly improved treatment outcome [6]. By using the tumour’s molecular signature, this RTOG trial set the tone for personalised treatment based on molecular features. While not an imaging marker, HPV is a clear example of a predictive indicator of HNC response to therapy.

Image-based, non-invasive assessment of hypoxia, proliferation and stemness are in current focus, using exogenous or endogenous molecular markers that have shown promise in the earlier days of predictive assays.

Today’s medical imaging techniques allow for a more detailed tumour assessment, owing to the plethora of biomarkers developed for specific tumour characteristics and also to the combined anatomical-functional methods that highlight individual tumour features. Molecular imaging can serve several purposes: firstly, to assist with the optimal treatment choice; secondly, to monitor tumour response during treatment and analyse the possible need for treatment adjustment and third, to evaluate post-treatment response.

**Figure 1:**
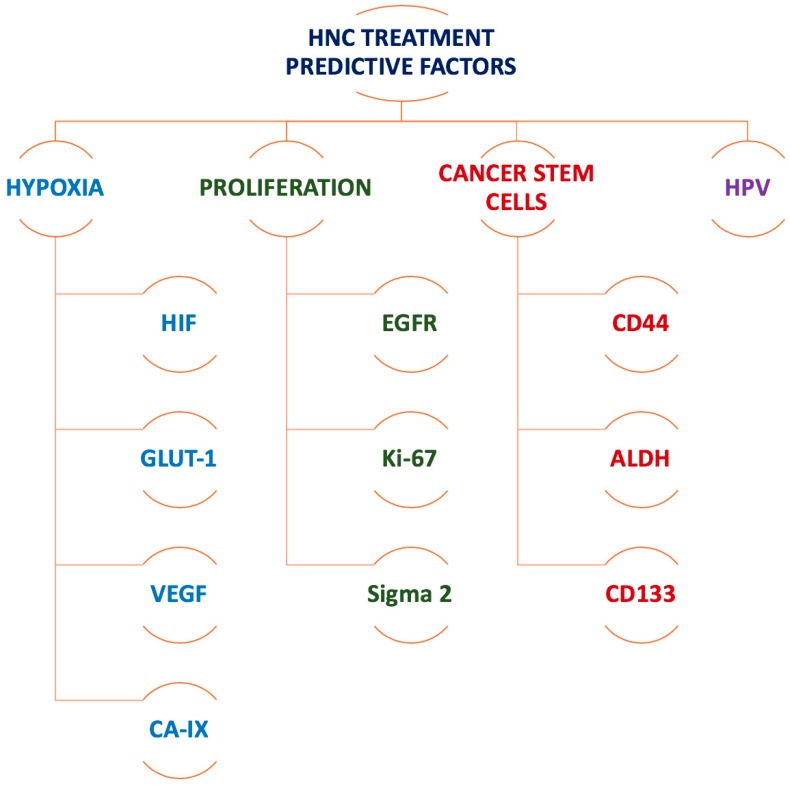
Summary of the most commonly investigated biomarkers that affect head and neck cancer (HNC) response to treatment. HPV, human papilloma virus; HIF, hypoxia inducible factor; GLUT-1, glucose transporter 1; VEGF, vascular endothelial growth factor; CA-IX, carbonic anhydrase IX; EGFR, epithelial growth factor receptor; Ki-67, cell proliferation-associated nuclear antigen; CD, cluster of differentiation molecule; ALDH, aldehyde dehydrogenase.

In this framework, molecular and functional imaging of tumour characteristics and signalling pathways are becoming increasingly important in the management of head and neck cancer. Positron emission tomography (PET), single photon emission computed tomography (SPECT) as well as functional magnetic resonance imaging (fMRI) all contribute towards the acquisition of specific molecular details that can guide treatment planning and optimally tailor treatment delivery. Most importantly, biomarkers should assist in patient stratification, in order to identify those patient groups that would most likely benefit from specific targeted therapies. Figure 1 presents a summary of the most commonly investigated biomarkers that affect HNC response to treatment.

The current work is an integrative review of current biomarkers for HNC used by various functional imaging techniques, aiming to add useful information to the existing literature and to highlight interdisciplinary fostering of developments in evidence-based radiation oncology.

## 2. Biomarker Identification by PET and SPECT

### 2.1. Imaging Biomarkers for Hypoxia

Tumour hypoxia is a leading cause of resistance to radiotherapy in head and neck cancer [7] and possibly the most studied tumour feature in HNC. Based on a recent analysis of PET studies employing various hypoxia markers, the incidence of hypoxia is about 75% per patient and 61% per tumour [8]. It is therefore not surprising that the inventory of hypoxia-specific biomarkers, is the most extensive when it comes to tumour characterisation.

Chronic hypoxia, which usually occurs in the tumour core due to limited oxygen diffusion, can be managed with reoxygenation through fractionated radiotherapy. On the other hand, acute hypoxia, that has oxygen perfusion limitation as the main cause, is a highly dynamic process requiring more attention. The character of acute hypoxia originates from the abnormal tumour vasculature, resulting from angiogenesis where newly formed blood vessels are often tortuous, displaying shunts, leakages and blocked passages that create a temporal state of hypoxia. The location, duration and/or propensity to temporal hypoxia within the tumour, are all unpredictable. Owing to this fact, biopsy samples are usually unreliable for the quantification of hypoxia. They are still used in combination with immunohistochemical methods, employing endogenous cell markers such as the hypoxia inducible factor (HIF-1), carbonic anhydrase IX (CA-IX), or exogenous markers for cytological coloration (with nitroimidazole compounds) [9]. A more direct technique, so far considered the optimal method for hypoxia detection, is the polarographic oxygen electrode that measures tissue oxygen pressures (pO2). Nevertheless, to overcome the invasiveness of the above-mentioned methods, the most accepted clinical solutions to quantitate hypoxia are the non-invasive functional imaging techniques that gain more room and trust.

In order to validate functional imaging as biomarkers for treatment response, it is important to analyse their correlation with hypoxia-specific molecular biomarkers, such as the ones mentioned above. Generally, expression of HIF-1, CA-IX or glucose transporter 1 (GLUT-1), is associated with poor outcome in head and neck cancer patients irrespective of the treatment provided [10]. In the paragraphs below, the most studied hypoxia-specific radiotracers are succinctly described while also specifying their correlation (where available) with biomarkers of tumour hypoxia.

The first hypoxia-specific in vivo imaging agent developed and tested in patients was fluoro-misonidazole (^18^F-MISO), being the most extensively used radiotracer today. The large number of studies and trials undertaken with F-MISO to test its safety, bio-distribution, uptake and hypoxia-specificity, highlight both promising results as well as limitations. While highly popular, F-MISO has not been established as a gold standard for hypoxia due to contradictory results regarding correlation between uptake and outcome, as well as its association with other tumour parameters (see Table 2). While Sato et al. [11] demonstrated a good correlation between F-MISO uptake and HIF-1α expression in oral squamous cell carcinomas, Norikane et al. [12] could only observe a weak association between the two parameters. This weak correlation could be due to the interference of several stimuli, other than hypoxia, that could lead to overexpression of the HIF-1α transcription factor [13].

A recent study reported by Löck et al. [22] showed the role of ^18^F-MISO as biomarker for selection of patients at high risk of locoregional recurrence after chemo-radiotherapy, demonstrating the importance of functional imaging in patient stratification based on treatment monitoring. The study that included two cohorts (exploration vs validation) showed that residual tumour hypoxia during chemo-radiotherapy is a strong determinant of resistance to treatment, as identified by second week FMISO-PET image quantification.

^18^F-fluoroazomycin-arabinofuranoside (^18^F-FAZA), a successor of F-MISO, is the next most popular PET radiotracer for hypoxic subvolumes, demonstrating a good prognostic role given the correlation between high tumour uptake and poor treatment outcome [23,24]. The DAHANCA 24 trial was conducted to investigate the prognostic value of F-FAZA in HNC patients treated with radiotherapy, finding a large inter-tumour variability in tracer uptake, which was associated with poor outcome in patients with hypoxic tumours [23]. The study found no correlation between hypoxia and HPV status. Nevertheless, non-smoking HPV positive patients had a more favourable prognosis, due to increased tumour radiosensitivity. This observation is in line with the results reported by other studies [25].

Patient stratification is a critical step towards individualised treatment and F-FAZA was shown to play an important role in the response assessment of HNC patients to hypoxic cytotoxins [24].

Advantage over F-MISO: Higher hydrophilicity and better clearance kinetics from oxic tissue; higher hypoxic contrast.

Presenting with good pharmacokinetics and high tumour uptake in head and neck cancer patients, ^18^F-fluoroery-thronitroimidazole (^18^F-FETNIM) was tested by a few groups for its hypoxia specificity, with a general conclusion that more work is needed to justify the use of F-FETNIM as substitute for F-MISO or F-FAZA. A more recent study evaluated the correlation between the expression of several tumour biomarkers and F-FETNIM uptake, including HIF-1, the VEGF and microvessel density (CD31) [17]. No correlation was found between F-FETNIM uptake and any hypoxia or blood flow (assessed with ^15^O-H_2_O) biomarker analysed. The expression of HIF-1α was found to fluctuate due to both hypoxia and reoxygenation, concluding that HIF-1α might not be a reliable hypoxia biomarker in clinical settings [17].

Advantage over F-MISO: More hydrophilic; higher tumour-to-background ratio.

The role of ^18^F-2-nitroimidazol-pentafluoropropyl acetamide (^18^F-EF5) in head and neck cancer patients was assessed by Komar et al. [18,26]. Their studies demonstrated a high tumour uptake of F-EF5 and a stronger correlation with treatment outcome than F-FDG, cataloguing EF5 as a possible surrogate marker for radioresistance. However, when trying to correlate it with blood flow markers, no statistically significant association was found between F-EF5 and the perfusion tracer ^15^O-H_2_O. HNC cell lines showed a very good correlation between CA-IX/HIF-1α expression and F-EF5 uptake, demonstrating the tracer affinity not only towards more hypoxic tumours but also towards an adverse, more aggressive phenotype [19]. Further clinical research might elucidate the genotype behind the studied phenotypes.

Advantage over F-MISO: More hydrophilic, with stable chemistry, however, only trivial advantage over more established radiotracers.

The feasibility of F-flortanidazole (^18^F-HX4) head and neck cancer was tested comparatively with F-MISO in twelve patients, showing several qualities: possibility for imaging within a shorter time period after injection (<2 h), high sensitivity and specificity and good correlation with CA-IX expression [20].

Advantage over F-MISO: Higher specificity, selectivity and faster clearance.

^64^Cu-diacetyl-bis(*N*^4^-methylthiosemicarbazone) (^64^Cu-ATSM) was investigated by several groups as an alternative to ^18^F-based agents, due to its high membrane permeability and low redox potential. Several other attributes determined testing ^64^Cu-ATSM in HNC patients, showing fast cellular diffusion and good tumour uptake with high tumour-to-background ratio after a short pre-scanning time [27]. Distribution in tumours was however, shown to be perfusion limited, thus not all hypoxic areas might be identified. The tracer showed high sensitivity in HNC but low specificity in predicting response to therapy [28]. Clinical studies are questioning the role of Cu-ATSM as a hypoxic marker due to conflicting evidence regarding its hypoxia selectivity. It is hypothesised that tracer retention in cells might not be fully due to hypoxia but influenced by the intracellular redox potential [29].

Advantage over F-MISO: None that would justify a real advantage.

Owing to its promise as a hypoxia-specific marker in animal models, ^124^I-iodoazomycin galactopyranoside (^124^I-IAZGP) was tested in 10 head and neck cancer patients to evaluate the safety, biodistribution and imaging features of this nitroimidazole compound [30]. The advantage over previously developed and radiotracers was considered to be the long half-life (4.2 days) which enables late PET imaging. While the tracer exhibited good clearance and no toxicity, the lack of differential uptake in any neoplastic lesion was an unexpected negative result. This was thought to be due to the low positron yield of ^124^I and also to dosimetric challenges imposed by the long-lived nuclide.

Advantage over F-MISO: Longer half-life due to ^124^I, which allows late PET imaging but not without other limitations.

SPECT imaging does not compete in popularity with PET imaging. While radiotracers normally used in SPECT have longer half-lives, allowing delayed scans, the spatial resolution of SPECT is inferior to PET which is an important factor when it comes to image analysis. The lower interest in SPECT and the smaller number of radionuclides developed have also left the aspect of tracer quantification behind. The number of studies investigating SPECT tracers for hypoxia detection is scarce and generally old, despite some promising results.

Iodoazomycin arabinoside (IAZA), a misonidazole analogue, has been labelled with ^123^I as well as with ^125^I to be trialled as hypoxia imaging biomarkers. In head and neck cancer patients ^123^I-IAZA identified tissue areas with impaired perfusion, thus indicating the presence of hypoxia [31]. However, no further studies have been performed to strengthen the role of IAZA in head and neck cancer response assessment to treatment.

Butylene amineoxime (^99m^Tc-HL91) a non-imidazole compound, has shown strong correlation between the GLUT-1 and tracer uptake in a rat model. Given that hypoxia upregulates GLUT expression, ^99m^Tc-HL91 was considered a potential candidate for hypoxia detection [21]. Later studies assessed the role of the tracer in the identification of tumour recurrence among HNC patients, without further investigations of its potential as a hypoxic marker [32].

While the arsenal of molecular markers for hypoxia imaging is increasing, the clinical focus should be on those that beside their cost effectiveness, also supply important information beyond that offered by the already established biomarkers. As shown in Table 2, there is also need for a better correlation between imaging and hypoxia-specific biomarkers to validate the efficacy of PET/SPECT radiotracers.

### 2.2. Imaging Biomarkers for Tumour Proliferation

Similar to hypoxia, PET imaging with proliferation kinetics-specific markers is the most common technique to evaluate and predict tumour proliferation and its association with treatment outcome. Several tissue markers have been identified to be responsible for tumour proliferation that represent suitable targets for functional imaging.

A key player in tumour proliferation is the epithelial growth factor receptor (EGFR) that was shown to be overexpressed in HNC and is linked to adverse outcome. Since cetuximab is a well-known EGFR inhibitor, van Dijk et al. [33] developed and investigated the effectiveness of an EGFR-specific tracer in mice with HNC xenografts. Autoradiography studies demonstrated high correlation between the PET tracer ^64^Cu-cetuximab-F(ab’)2 and EGFR expression, showing the tracer’s potential to monitor tumour response to EGFR-inhibitor treatment. The same group has previously investigated SPECT imaging of tumour proliferation in HNC using ^111^In labelled with cetuximab F(ab’)2 fragments, showing good tracer uptake 4 h post-injection and optimal imaging 24 h after injection, indicating the potential effectiveness of EGFR inhibitors [34].

Recent SPECT (^177^Lu-PCTA-cetuximab) and PET (^64^Cu-PCTA-cetuximab) imaging indicated the suitability of these agents to be used for specific targeting of tumours that express high levels of EGFR and, furthermore, the potential for radioimmunotherapy using the same radionuclides in cetuximab-resistant tumours [35].

One of the most widespread proliferation-specific radioisotopes used in PET is ^18^F-FLT (3’-Deoxy-3′[18F]-fluorothymidine), an analogue of thymidine that is phosphorylated by the key enzyme in DNA synthesis-thymidine kinase 1 (TK-1). Tumour uptake of ^18^F-FLT was shown to be well correlated with proliferation kinetics owing to the fact that the activity of TK-1 is elevated during the S phase [36,37,38].

The results of a kinetic analysis involving ^18^F-FLT in seven HNC patients, that underwent 5 days’ radiotherapy and one cycle of chemotherapy, was reported by Menda et al. [36]. Pre-treatment ^18^F-FLT tumour uptake was significantly reduced after treatment, which aligned with a decrease in thymidine kinase activity. The same tracer was employed by Troost et al. [37] for a group of ten oropharyngeal carcinoma patients in order to identify tumour sub-volumes with high proliferation. To monitor treatment response, patients underwent serial PET scans at baseline and during radiotherapy, showing a greater than two-fold decrease in ^18^F-FLT uptake in the initial treatment phase, which continued to decrease by the fourth treatment week. The identification of residual ^18^F-FLT sub-volumes enabled dose escalation for a better locoregional control [37]. In line with the above results, Hoeben et al. [39] concluded that a change in ^18^F-FLT uptake early during treatment is a strong indicator of long-term outcome and a valuable guide for individualized patient management. This conclusion was based on a study that accrued 48 HNC patients imaged with sequential PET for treatment response monitoring before treatment, during the second and fourth weeks of radio-chemotherapy.

Research into biomarker imaging has identified sigma-2 receptors as potential candidates for the identification of proliferative tumour sub-volumes in HNC, based on the elevated expressions of sigma receptors in proliferating cells compared to quiescent cells [40]. PET studies with radiolabelled sigma-2 receptor ligands supplied superior tumour specific information compared to thymidine kinase-1 based radiotracer imaging [41,42]. Among the tested radioisotopes was ^11^C (limited clinical utility due to a short 20.38 min half-life), ^18^F and ^76^Br [40,41]. In a comparative study between ^76^Br-labelled ligands with the more established ^18^F-FLT, Rowland et al. [41] reported a better tumour visualization with ^76^Br, given by a 9 times higher tumour-to-normal tissue ratio 2 h post injection, followed by a faster metabolic clearance of non-specifically bound ^76^Br compounds. Further studies are needed to confirm these results and to justify the use of ^76^Br as a PET agent.

A promising ^18^F-labeled σ2-receptor ligand is ^18^F-ISO-1 which was already trialled in a pilot study involving head and neck cancer patients [40]. The tracer showed significant correlation between ^18^F-ISO-1 uptake and the marker protein Ki-67—a cell proliferation marker existent in the nuclei of cycling cells-only. The results allowed for patient stratification using the Ki-67 value as a threshold to define high proliferative status (Ki-67 > 35%) and low proliferative status to serve for further treatment adaptation [43].

## 3. Biomarker Identification by fMRI

Functional MRI is uniquely placed to support chemoradiotherapy for HNC where repeated investigations can be performed without additional ionising radiation to the patient. Given the mixed and complex anatomy of the head and neck, it is ideally suited to cancer staging and follow up by virtue of superior soft tissue imaging [44]. Moreover, versatility in the use of different gradients, pulse sequences and contrast agents, facilitates the imaging of tissue and cellular characteristics as well as indicative parameters in tumour physiology. Magnetic resonance spectroscopy (MRS) takes a different approach again to identify and measure molecular and metabolic constituents in selected regions of interest [45]. Table 3 lists frequently used MR approaches to imaging biomarkers in HNC.

As discussed above, hypoxia presents a significant obstacle in treating HNCs and much emphasis in using fMRI has been directed toward its localisation and quantification in terms of perfusion and diffusion [44,54]. Diffusion Weighted Imaging (DWI) exploits differences in the Brownian motion of interstitial fluid in normal tissue and areas of restricted diffusion such as a tumour’s dense cellular architecture [55]. By applying different gradient durations and amplitudes (b values) the apparent diffusion coefficient (ADC) is calculated which has been used for differentiation of malignant and begin lesions, detection of sub-centimetre metastatic lesions and is reported to be an independent prognostic factor for HNC [47,48,56].

Dynamic contrast-enhanced (DCE) MRI images tissue perfusion using gadolinium contrast. The aberrant microvasculature from malignant angiogenesis is assessed from a rapid series of sequential images (~2 s) tracking the elevated signal of gadolinium as it enters interstitial space from the capillary beds and then drained by tumour veins [44]. The temporal imaging is used to develop a concentration-time curve from which several parameter values are derived. One of the most commonly reported, K^trans^, is a constant of the intravascular efflux of contrast into tumoural interstitial space which has shown a strong predictive association with progress free and overall survival in advanced HNCs [57,58].

Blood oxygenation level dependent imaging (BOLD), uses the differing magnetic states of haemoglobin when oxygenated or deoxygenated, to determine states of acute hypoxia. Paramagnetic deoxyhaemoglobin induces localised dephasing and a shortening of the transverse relaxation time in T2*. To differentiate this effect from the iron content in static tissue, the transverse relaxation rate (R2*) is considered here, were R2* = 1/T2* and an elevated R2* is indicative of hypoxia [46,59]. Being dependent on the presence of haemoglobin, this is an examination of tumour perfusion and as such, BOLD has been used to assess patient tumour oxygenation response to carbogen breathing and individual suitability for this anti-hypoxia treatment in radiotherapy [60].

MRS differs from MRI where instead of anatomical and physiological imaging, water proton signal is suppressed in favour of signal from low concentration metabolites in a region of interest. Depicted as line charts, these show the intensity of signal for specific metabolites [59]. Choline is a product of the metabolism of cell membranes and detection of elevated levels is indicative of heightened cellular proliferation by increased membrane turnover or synthesis. When its measure is taken as a ratio with creatine (choline/creatine), elevations in this ratio have been shown to be a strong indicator of high grade neoplasm. High signal from membrane lipids by MRS can be the result of cellular destruction by necrosis and necrotic tissue may also show a peak in lactate methyl as a result of anaerobic metabolism. MRS has also demonstrated an ability to differentiate between normal tissue and HNC by elevated choline/creatine ratios as well as assist predictions of therapeutic response [53]. A study of 46 patients with HNC found persisting choline levels post treatment was a strong predictor for residual cancer [52].

The diversity of possible approaches in fMRI and MAS, comparatively small numbers of study participants and a lack standardisation among the methods thus far, are limiting factors on validity and the possible comparison between studies to date [47]. Nevertheless, the studies show significant potential for the identification of biomarkers and their correlation with outcomes where the multiplicity of imaging approaches from this single modality offers potential to characterise biomarker expression in an individual’s tumour and track its response during and post treatment.

## 4. The Road Ahead: Imaging Biomarkers for Cancer Stem Cells

Cancer stem cells (CSC) are a subpopulation of tumour cells that display a series of features that distinguish them from other cancer cells. CSCs have the ability to proliferate indefinitely while creating all lineages of the original tumour, demonstrate higher resistance to treatment than non-stem cancer cells which could be linked to their predominantly quiescent nature and additionally, exhibit enhanced DNA repair ability and a high level of cellular plasticity [61].

The first qualitative study on the identification of CSCs in head and neck carcinomas was reported by Prince et al. [62], after isolating a subpopulation of tumour cells with stem-like properties. Subsequently, other reports in the scientific literature have demonstrated the existence of CSCs in HNC, although in varying proportions [63,64]. The reason for variations among CSC subpopulation is multifactorial and depends on the cell line used for the study, the markers employed for CSC identification and the interpretation of the findings.

For the identification of CSCs, cell surface markers are typically used such as cluster of differentiation (CD) molecules, which are surface proteins that enable the analysis of cell differentiation. The hyaluronic acid receptor CD44 was found suitable for CSC identification in malignancies of epithelial origin, being overexpressed in a number of cancers [65]. For several anatomical sites related to HNC, high expressions of CD44 were associated with invasiveness, distant metastases and treatment resistance [66]. Furthermore, Ilardi et al. identified a strong correlation between CD44 and CA-IX overexpression in a series of tongue cancers sorted by grading, staging and biological behaviour [67]. Stromal CA-IX expression was associated with the occurrence of adverse events, which was thought to be linked to CA-IX-mediated mesenchymal changes that trigger tumour invasion [67]. These findings strengthen the need for the development of CA-IX targeting agents in order to block the multiple transduction signals that underlie treatment resistance of both hypoxic as well as cancer stem cells.

Cells that express the intracellular enzyme aldehyde dehydrogenase (ALDH) were found to be a subset of the CD44+ cells and the activity of ALDH on its own was shown to be a highly selective marker for CSCs [68]. Recent studies on HNC revealed that cells exhibiting ALDH+ preserve their tumorigenic abilities after irradiation and can trigger tumour repopulation [69]. The cell surface glycoprotein CD133 (also known as AC133) is another CSC marker based on the fact that its overexpression in HNC is negatively correlated with survival, also serving as an indicator of tumour repopulation and malignant progression [70].

The particular properties of CSCs can now be explored with various imaging techniques, PET and MRI being the most suitable for treatment response assessment and the evaluation of metastatic spread [50]. However, CSC imaging is not without challenges, as the chosen techniques must be sensitive enough to detect these rare cells within a large population of tumour cells. Still, a number of in vitro and in vivo studies have tested CSC-specific PET/SPECT radiotracers. The CD133 surface glycoprotein was explored via ^64^Cu-ATSM PET in mouse colon carcinoma [71]. The imaging results showed good tracer accumulation in rich areas of CD133+, thus rendering the radionuclide as a potential imaging agent for CSCs. This result could also be due to the affinity of ^64^Cu-ATSM towards hypoxic regions, given that the CD133+ cells were also under hypoxic conditions. Gaedicke et al. investigated two xenografted cell lines that overexpressed CD133 using ^64^Cu-NOTA-AC133 as PET imaging agent, showing a clear difference in invasiveness between the two xenografts [72]. With a good tumour-to-background contrast, the intensity of the PET signal reflected the microscopic pattern of AC133+ expression. 

To date, the number of in vivo studies investigating molecular imaging of CSCs in HNC is very scarce. Spiegelberg et al. explored the role of CD44 imaging in prediction and response monitoring after treatment with the AT13387 radiosensitiser of a squamous cell carcinoma mouse model [73]. For this purpose, PET imaging with ^124^I-radiolabelled anti-CD44v6 AbD19384 was undertaken in comparison with standard ^18^F-FDG. While FDG could not reveal any difference in receptor expression between the treated and untreated mice, ^124^I-AbD19384 successfully identified the tumours with high expression of CD44. However, treatment monitoring showed that the radiosensitiser AT13387 had no success in reducing the expression levels of CD44 in the treated tumour.

Still in exploratory stages, superparamagnetic iron oxide nanoparticles for MRI have been used to image CSCs where the elevated expression of CD44 is indicative of stemness [50]. Hyaluronic acid, a natural ligand for CD44 has been conjugated with magnetic nanocrystals to be selective of cells overexpressing this marker and used in in vitro and in vivo studies. An animal study looking at CD44 overexpression as stemness in breast cancer, found these conjugates were able to identify CD44+ cells where T2 signal was decreased by the magnetic nanoclusters in contrast with other tissue [51].

While yet in its infancy, molecular imaging of CSCs is a promising area for clinical research that needs further investigation in a broader tumour spectrum.

## 5. Conclusions

Treatment response monitoring and adaptation using functional imaging techniques is a relatively new area of research that is yet to be routinely implemented into clinics. While the number of biomarkers and radiotracers designed for PET/SPECT and MR imaging is continuously increasing, there is still room for improvement concerning their specificity, selectivity, reproducibility and overall efficiency for treatment guidance. It is also important for novel molecular biomarkers to supply information beyond that offered by the already established agents.

Quantitative imaging of biomarkers is a prerequisite for accurate treatment response evaluation and should represent the next step towards personalised treatment together with standardisation of imaging protocols to facilitate the creation of imaging databases. Advances in radiomics already allow for the interpretation of quantitative features in head and neck cancer patients. This is a very important aspect of personalised therapy, considering the complex anatomical structures, significant inter-patient variations and large tumour heterogeneities characteristic of HNC. Recent developments in radiomics for HNC show promise in pathologic classification, risk stratification and longitudinal monitoring of treatment response in normal tissue, though not without challenges that include requirements for large data processing, reproducibility, evaluation of radiogenomic association and standardised reporting protocols [74].

Despite the large number of available biomarkers, no gold standards have been yet established, as none can reflect the characteristics of the tumour as a whole. A combination of biomarkers could possibly serve this purpose better, though more work is needed to validate the efficacy of combined imaging and tissue markers in order to increase treatment effectiveness.

## Figures and Tables

**Table 1:** Predictive assays for tumour response to radiotherapy and their limitations (modified from [5]).

Predictive Assay	Oxygenation Status	Proliferative Potential	Intrinsic Radioresistance (Subpopulation of Cancer Stem Cells?)
**Purpose**	To identify the patient group that would benefit from hypoxic cell sensitisers.	To differentiate between tumours with slow and fast proliferation.	To correlate cell line radiosensitivity with tumour response to radiation.
**Technique**	Polarographic needle electrode Endogenous/exogenous markers; 3D models; microvessel density.	Kinetic parameter measurements: length of S phase, potential doubling time; labelling index; clonogenic survival.	Dose-response curves; Colony growth (MTT), micronucleus, chromosomal, DNA damage (Comet) assays; tumour control assay.
**Limitation**	Invasive; Unreliable (biopsies); Costly and time consuming; Require high level expertise.	No robust correlation between kinetic parameters and treatment outcome; Time consuming.	Highly time consuming.
**Present/Future**	Hypoxia-specific PET radiotracers: F-MISO; F-FAZA; Cu-ATSM; other radiotracers BOLD/TOLD (blood/tissue oxygen level-dependent) MRI	Proliferation-specific PET radiotracers: F-FLT; F-ISO-1; ^11^C-based radiotracers.	Cancer stem cell-specific PET radiotracers; MRI; HPV-status based identification of more radioresponsive tumours.

**Table 2:** Correlation between PET/SPECT tracers and hypoxic tumour markers/parameters.

PET STUDIES		
Tracer	Tumour Marker/Parameter for Hypoxia	Correlation between PET Tracer and Tumour Markers
^18^F-FMISO	pO2 (Mortensen 2010) [14]	No correlation was observed between pO2 measurements (Eppendorf) and F-MISO. Tumours were more hypoxic based on pO2 measurements.
	HIF-1α (Sato 2013) [11]	Strong correlation with HIF-1α was found.
	HIF-1α (Norikane 2014) [12]	Only a weak correlation of hypoxic volume with HIF-1α expression was observed.
	CA-IX (Bittner 2016) [10]	No correlation between CA-IX and tracer uptake was observed.
^18^F-FAZA	Blood flow via ^15^O-H_2_O (Shi 2010) (compartmental model analysis) [15]	Very similar distribution pattern between tracer accumulation and blood flow during early imaging and different pattern at later imaging times in line with tracer uptake by hypoxic regions.
^18^F-FETNIM	pO2 (Lehtiö 2004) [16]	Correlation between the hypoxic volume as indicated by F-FETNIM and pO2 was only found in a limited number of patients.
	HIF-1α, VEGF, CD31 Blood flow via ^15^O-H_2_O (Grönroos 2014) [17]	Immunohistochemical biomarkers for hypoxia and blood flow did not correlate with F-FETNIM uptake.
^18^F-EF5	Blood flow via ^15^O-H_2_O (Komar 2014) [18]	No correlation between F-EF5 uptake and blood flow assessed with the perfusion tracer ^15^O-H_2_O
	CA-IX, HIF-1α (cell line study) (Silén 2014) [19]	Very good correlation between F-EF5 uptake and CA-IX/HIF-1α expressions, indicative of a more aggressive phenotype.
^18^F-HX4	CA-IX (Chen 2012) [20]	F-HX4 uptake is correlated with CA-IX expression.
^64^Cu-ATSM		No correlation studies reported
**SPECT STUDIES**		
^123^I-IAZA		No correlation studies reported
^99m^Tc-HL91	GLUT-1 (in rat tumour) (Yutani 1999) [21]	Strong expression of GLUT-1 in tumour sites with high tracer uptake, showing hypoxia-avid properties.

**Table 3:** MRI imaging techniques for biomarkers in HNC.

Name	Biomarker	Pulse Sequence	Contrast	Notes	Reference
**BOLD** Blood oxygen level dependent imaging	Acute hypoxia	Multi-echo GRE	None	Measured by R2*	Padhani et al. (2007) [46]
**DWI** Diffusion weighted imaging	Tissue diffusion Chronic hypoxia	Echo planar imaging Single shot spin echo	None	Measures diffusion restriction resulting from cellular density of tumour tissue	Jansen et al. (2016) [47], Lambrecht et al. (2014) [48]
**DCE** Dynamic contrast-enhanced imaging	Angiogenesis Tissue perfusion Hypoxia	Fast multiphase spoiled GRE	Gadolinium	Sequential imaging measures movement of contrast from tumour vasculature to interstitial space	Bernstein et al. (2014) [44]
**ASL** Arterial Spin Labelling	Tumour perfusion	Echo planar imaging Multi shot spin echo	None	Radiofrequency waves magnetically label arterial blood water for tracking	Detre et al. (2012) [49], Jansen et al. (2016) [47]
**Cancer Stem Cell Imaging**	CSCs (CD44+)	T2	SPIO (USPIO)	CD44+ cells T2 signal decreased by magnetic nanoclusters	Heryanto et al. (2014) [50], Lim et al. (2011) [51]
**MR Spectroscopy**	Metabolite concentration		None	Biochemical rather than anatomical information	King et al. (2010) [52], Razek & Poptani (2013) [53]

GRE, Gradient recalled echo sequence; SPIO, Superparamagnetic Iron Oxide; USPIO, Ultra-small Superparamagnetic Iron Oxide; R2*, the relaxation rate of T2*.
